# Syntactic theory is also a metaphor

**DOI:** 10.3389/fpsyg.2014.01554

**Published:** 2015-01-08

**Authors:** Tommi Leung

**Affiliations:** Department of Linguistics, United Arab Emirates UniversityAl Ain, United Arab Emirates

**Keywords:** syntax, action, syntax-action analog, metaphor, biolinguistics, granularity mismatch problem

Pulvermüller (2013) and Moro (2014a,b) exchange views on the possible relation between syntax and action. Pulvermüller points out that human's action displays hierarchical and embedding properties, analogous to how a sentence is organized by the human mind. His claim is that syntax may not be as sui generis as syntacticians upheld. As more evidence reveals the neurobiological mechanisms underlying animals' actions and perceptions (Pulvermüller and Fadiga, 2010), Pulvermüller expects that mechanisms which describe the animal combinatorial capacity for action can find a meaningful counterpart in syntax. Indeed, this alleged parallelism between language and other cognitive domains has been receiving wider support as the field of Biolinguistics began to take shape (Hauser et al., 2002; Patel, 2008; Knott, 2012; Boeckx and Fujita, 2014). On the contrary, Moro doubts that the syntax-action analog is at best a metaphor. His opposition stems from the observation that functional categories as the major building blocks of syntax are unattested in action. Moreover, Merge and Move (Chomsky, 1995) as two primitive operations giving rise to the hierarchical structure of sentence (in particular form, such as the X-bar schema), are hardly instantiated in action. Since other syntactic observations such as Locality and Island Constraints (Ross, 1967; Manzini, 1992) are parasitic on Merge/Move (though they may be considered extraneous to syntax, e.g., Boeckx, 2012), neither Merge/Move can be realized in the mental representation of action planning.

It is fair to say that Pulvermüller's proposal for the action-syntax analog in full scale, while promising in its spirit (esp. from a biolinguistic perspective, e.g., Boeckx and Fujita, 2014), is not rigorously argued, given the elusive relation between action and syntax which underlies the inquiry. As Moro correctly points out, actions are subject to physical conditions, whereas syntax is part of mental representation. Even if action planning is considered instead, it is constrained by how the theory of spatial memory interacts with the environment and situation. Pulvermüller's description of a daily routine of someone walking into the bathroom, opening the bathroom light, walking to the basin, picking up the toothbrush and brushing his teeth, etc., represents a fixed sequence of events stored in the long-term procedural memory. This procedure is incomparable with online sentence processing which requires continuous access to working memory, and not to mention, the theory of syntax as implicit knowledge. Pulvermüller's cherry-picked example is therefore misleading.

However, Pulvermüller's misconception is not unique, as it stems from linguists' oblivion of the *granularity mismatch problem* (GMP) (Poeppel and Embick, 2005) and, moreover, what the current syntactic theory offers. On the other hand, Moro's conviction that syntax of action is a metaphor does not add any scientific value to the discussion, since the operations in syntactic theory are also metaphorical. I am doubtful whether syntacticians agree on the foundation of Merge/Move, or simply employ them out of fashion. While Merge/Move are theoretical constructs, speakers do not virtually “merge” (i.e., “to combine or to join together”) grammatical objects to form a constituent in the mental representation, or “move” (i.e., “to change the position of”) a word from one position to another (e.g., in the derivation of wh-questions). Merge/Move are merely metaphorical expressions which offer one particular vision to describe the derivation/representation of a sentence. One can simply discard Merge/Move and propose, for instance, a generalized mapping theory of syntax without any loss of descriptive power. For instance, instead of postulating the operation Merge (*the, cat*), one can say that “the” maps with a position *Det* which is structurally adjacent to another position *N* which is further mapped by “cat,” and the two positions constitute a conceptual grouping. If an element is mapped with two (or more) positions in the same computational space, it underlies the concept of Move. Further generalizing this, syntactic computation can be reduced to a list of: (i) concept-bearing linguistic objects, (ii) grammatical positions, (iii) mapping relations between objects and positions, (iv) instructions for conceptual grouping. To illustrate, consider the wh-question “*who did John see*?” (i) consists of the set {*who, did, John, see*}, (ii) consists of the set of positions {*N_1_, T, N_2_, V*}, (iii) consists of the set of ordered mappings {(*who, N_1_, did, T, John, N_2_, see, V*)}, and (iv) instructs that {*N_1_, T*}, {*N_1_, V*}, {*N_2_, V*}, and {*T, V*} are the set of conceptual groupings. Note that the conceptual grouping {*N_1_, T*}, {*N_1_, V*} is tantamount to saying that “who” moves from the base position to the sentence-initial position. Assuming that (i–iv) underlie syntactic computation, we will understand that not only Merge/Move, but also tree diagrams, are metaphorical and can be deconstructed. Indeed, once we start practicing trees, we are constrained by a single perspective of analysis, and an alternative scenario is missed. Whether we are able to generalize from the plethora of metaphors in syntax relies heavily on the academic upbringing. As a mentor once educated me, syntax can be done without resort to trees, as the tree-drawing practice inevitably incorporates the input-output metaphor (Boland, 2005). There are definitely virtues of using trees and terms like Merge/Move as syntax merits a lingua franca. However, these operations may not be translatable in other cognitive domains. To solve the puzzle of GMP in the inquiry of syntax-action analog, one would need to restudy or reduce sui generis operations to domain-generic concepts.

To conclude, the GMP needs to be resolved before the syntax-action analog turns out to be a just-so story. For cognitive psychologists such as Pulvermüller, it would be more desirable to focus on nature of hierarchy and chunking in serially ordered action plans (e.g., Lashley, 1951; Rosenbaum et al., 1983, 2007). If the syntax-action analogy must be seriously entertained, one can demonstrate how the perception/production theory of action (Hommel et al., 2001) is analogous to that of sentence comprehension/production. On the other hand, the nature of Merge/Move and syntactic derivation remains the job of armchair linguists. As long as Merge/Move are metaphorical, GMP remains, and whether they are indeed instantiated in human action becomes a largely vacuous inquiry.

## Conflict of interest statement

The author declares that the research was conducted in the absence of any commercial or financial relationships that could be construed as a potential conflict of interest.
